# Specific Strains of Honeybee Gut *Lactobacillus* Stimulate Host Immune System to Protect against Pathogenic Hafnia alvei

**DOI:** 10.1128/spectrum.01896-21

**Published:** 2022-01-05

**Authors:** Haoyu Lang, Huijuan Duan, Jieni Wang, Wenhao Zhang, Jun Guo, Xue Zhang, Xiaosong Hu, Hao Zheng

**Affiliations:** a College of Food Science and Nutritional Engineering, China Agricultural University, Beijing, China; b Faculty of Agriculture and Food, Kunming University of Science and Technology, Kunming, Yunnan, China; c Faculty of Life Science and Technology, Kunming University of Science and Technology, Kunming, Yunnan, China; d College of Plant Protection, China Agricultural University, Beijing, China; Chinese Academy of Sciences

**Keywords:** honeybees, antimicrobial responses, microbiome, innate immunity, symbiosis, colonization resistance, immune priming, S-layer protein, *Hafnia alvei*, antimicrobial peptides, gut microbiota, immune response

## Abstract

Honeybee gut microbiota plays an important role in host physiology and metabolism. Recent studies have shown that the influence of the resident microorganisms in the regulation of honeybee immune system is profound, which protects against the pathogen Serratia marcescens. However, only few of the core gut members in the regulation of immune functions have been studied. Here, we explored how different bee gut bacterial species aided in the clearance of the pathogenic Hafnia alvei, which causes bee septicemia with a high mortality rate. We found that both Gilliamella apicola W8136 and Lactobacillus apis W8172 protect honeybees from the opportunistic pathogen, while two other strains from *Gilliamella* and *Lactobacillus* did not affect the invasion of *H. alvei*. Transcriptomic analysis revealed that gut species induced different expression profiles in the gut. Specifically, two regulator genes from the Toll pathway, PGRP-S3 recognizing Gram-positive and Spätzle that bind to the Toll protein for the downstream signal transduction, were elevated by *L. apis*. Correspondingly, multiple genes encoding antibacterial proteins were also stimulated by *L. apis*. Interestingly, we found an increased expression of apidaecin, which also exhibited a high *in vitro* inhibitory effect on *H. alvei*. To elucidate the difference of strains in the host’s immune regulation, comparative genomic analyses indicate that the S-layer proteins unique to *L. apis* are potentially involved in honeybee Toll signaling and the activation of antibacterial protein production.

**IMPORTANCE** Honeybees are essential pollinators supporting global agricultural economies and food supplies. Recent honeybee decline has been linked to several factors, while pathogen infection is considered one of the most significant contributing factors. Although a limited number of bacterial pathogens have been identified, Hafnia alvei is one of the pathogens causing septicemia in adult bees. In this study, we showed that two bee gut members, *Gilliamella* and *Lactobacillus*, can clear H. alvei from invasion. Mono-colonization of specific strains can stimulate the host Toll signaling pathway and the downstream expression of AMPs. Specifically, apidaecin upregulated by the gut symbionts is more effective against the pathogen. Moreover, our genomic analysis suggests that the surface-layer proteins specific to *Lactobacillus* strains are an important driver of Toll signaling, highlighting the variation of bee gut strains in regulating the host immune system.

## INTRODUCTION

Honeybees (Apis mellifera) are important crop pollinators worldwide, which support the agricultural economy and international food supply. In recent years, on a global scale, a phenomenon called Colony Collapse Disorder has threatened honeybee health (1). The decline of honeybee colonies arises from multiple factors, including climate change, overuse of pesticides, and pathogen infections (2, 3). Honeybees are susceptible to a variety of pathogens and pests, such as bacteria (4), fungi (5), Varroa destructor (6), and hive beetles (7). Several bacterial pathogens have been identified hazardous to honeybees, and they mainly attack bee larvae. Paenibacillus larvae and Melissococcus plutonius cause highly contagious bacterial diseases of honeybee brood (8, 9). Recently, it has been found that bacterial pathogens can infect adult bees, though this may be underrecognized due to bees’ social immunity mechanisms (10). For example, S. marcescens is a widespread opportunistic pathogen of adult bees and is highly virulent under conditions such as perturbation of the normal gut microbiota (11).

Hafnia alvei is an opportunistic pathogen of plants and animals and has been detected in the guts of honeybee workers (12). It was first isolated by Bahr in 1919 (previously named Paratyphus alvei) as a specific pathogen in bees (see Ref. 13). Experimental infection by injection of H. alvei into the chest of honeybees causes septicemia with a mortality rate of over 90%, and H. alvei could be isolated from the digestive tract and tissues of dead bees (reviewed in Ref. 14). H. alvei was also detected from bee hemolymph, guts, and honey from unhealthy hives, suggesting it is a potential agent in septicemic infection in bees. Moreover, H. alvei and P. larvae both show higher abundance in the guts of bees from colonies exhibiting American foulbrood (AFB) symptoms, suggesting that H. alvei is a secondary invader associated with AFB (12). Genomic and phenotypic analysis reveals that H. alvei harbors genes encoding bacteriocin and siderophore and exhibits antimicrobial activity toward *Bacillus* sp., suggesting its interactions with other gut microbes (15).

One major benefit that the intestinal microbiota can provide to its host is the protection against pathogen infection. The composition of gut microbiota of A. mellifera is simple, which comprises only few bacterial genera that probably have long evolutionary associations with their host (16). Compositional surveys revealed that the honeybee gut microbiota typically contains five core members (17), two genera of lactic acid bacteria, *Lactobacillus* Firm-5 and *Bombilactobacillus* (*Lactobacillus* Firm-4), *Gilliamella*, *Snodgrassella*, and *Bifidobacterium*. This distinctive gut community plays an important role in bee physiology, nutrition metabolism, and immune functions (18).

Honeybee antibacterial immunity relies on the Toll and Imd pathways, which primarily regulate the production of AMPs released into circulation under the activation of the two signal pathways during pathogen infection (19). In honeybee, four families of AMPs—abaecin, apidaecin, defensin, and hymenoptaecin—have been recognized, which are key components of the innate immune system of honeybee (20). So far, several studies have shown that the gut microbiota can influence bee health by the modulation of host immune responses. When the bees colonized with gut microbiota and mono-colonized with one of the core members, Snodgrassella alvi, the gene expression of apidaecin and hymenoptaecin was stimulated in the gut (21). The concentration of apidaecin was elevated in both gut tissue and hemolymph, which facilitated the clearing of E. coli invasion in the hemolymph. In addition, colonization with both live and heat-killed S. alvi leads to the upregulation of AMPs and protects host from the infection of pathogenic S. marcescens. Although researchers also investigated the roles of *Lactobacillus* in the protection against bee pathogens, they always targeted non-core gut members (e.g., Apilactobacillus kunkeei), inhibiting nectar and hive materials other than the gut and even environmental bacterial species (22, 23). When the normal bee gut community is perturbed by antibiotic or herbicide, the relative abundance and diversity of core bacterial taxa are depressed (24). The mortality after the challenge with S. marcescens was dramatically elevated (11, 25). All these findings indicate that the honeybee gut microbiota plays an important role in immune function and protection against microbial pathogens. Thus, honeybee with a simple and specific gut community provides an enticing model system for studying host-microbe interactions and the mechanisms by which gut symbionts influence host immune system (26). Moreover, all core bacterial genera contain multiple closely related species coexisting in honeybee gut, and they show high strain-level diversity in metabolic functions (27). Therefore, investigations on the other gut members will provide further insight into the mechanisms underlying the regulation of host immune system by gut symbionts.

In this study, we characterized the pathogenicity of a *H. alvei* strain isolated from the guts of honeybee. *In vivo* test confirmed that *H. alvei* is pathogenic in worker bees, particularly those with disturbed gut microbiota by antibiotic treatment. Then, we investigated the influence of honeybee’s native gut members on the host’s immune functions. Specifically, *Gilliamella* and *Lactobacillus* affect the expression of genes related to immune system and amino acid metabolism in the gut. Interestingly, both *Gilliamella* and *Lactobacillus* showed strain-level variation in the clearance of bacterial pathogen and the regulation of host AMP gene expression. Stain W8172 of *Lactobacillus* particularly stimulated the expression of apidaecin, and accordingly, *in vitro* assays showed that *H. alvei* is more susceptible to the two isoforms of apidaecin. To elucidate the mechanism underlying the difference of strains in host’s immune regulation, our comparative genomic analyses indicated the unique S-layer proteins are potentially involved in the PGRP-SA recognition and subsequently induce host immune Toll signaling pathway.

## RESULTS

### H. alvei is an opportunistic pathogen of honeybees.

**FIG 1:**
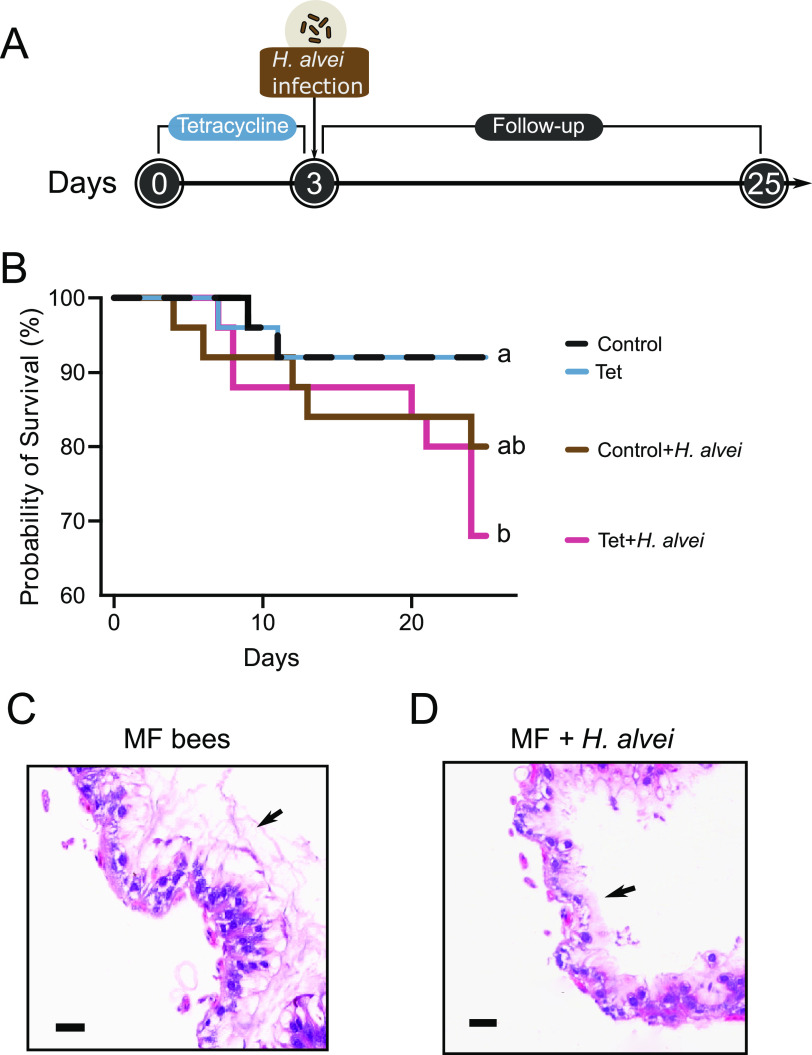
Tetracycline treatment reduced survivorship of honeybees exposed to *H. alvei*. (A) Schematic illustration of the experimental design. Groups of honeybees were treated with tetracycline and then were orally exposed to H. alvei. (B) Survivorship of control and tetracycline-treated honeybees alive after exposure to *H. alvei*. When challenged with H. alvei, bees without antibiotic treatment survived better than bees with disturbed microbiota by tetracycline administration in the diet. Total N = 30 bees from three replicate experiments for each treatment group. Letters next to the lines indicate significant differences in survivorship. (*P < *0.05, Mantel-Cox test) (C, D). Histopathologic evaluation with hematoxylin and eosin staining showed more prominent inflammatory changes in the epithelium of rectum of H. alvei infected group (C) than in that of MF group (D). Bars = 50 μm. Arrows indicate the thickness of epithelia.

Using *in vivo* virulence assays, we first explored whether H. alvei is pathogenic to adult honeybees. One H. alvei strain SMH01 was isolated from the worker bee gut. Then we investigated if the strain contributes to increased mortality of bees with normal or disturbed gut microbiota. Adult worker bees were collected from a brood frame in the hive and were treated with or without tetracycline. After introduction in the lab, groups of bees were fed filter-sterilized sucrose syrup (controls) or tetracycline suspended in the diet (Tet) for 5 days (Fig. 1A). Then we challenged bees with H. alvei SMH01. Bees were orally exposed to *H. alvei* in their food for 24 h. While strain SMH01 only moderately altered the survivorship of the control bees, the exposure to H. alvei significantly decreased the survival rate of Tet-treated bees (Fig. 1B). This indicates that H. alvei is opportunistically pathogenic to the susceptible host with altered microbiota composition and a possibly weakened immune system (10). To confirm the pathogenicity of H. alvei to honeybees, we performed histology of honeybee guts exposed to H. alvei. Accordingly, histopathological analysis of rectum tissue found significantly increased inflammation compared with microbiota-free (MF) bees individuals at Day 7 after the infection. Damaged tissue presented barely preserved mucosal architecture of the rectum in H. alvei-infected bees (Fig. 1C and D).

### Gut bacteria aids in the clearance of the pathogenic H. alvei.

**FIG 2:**
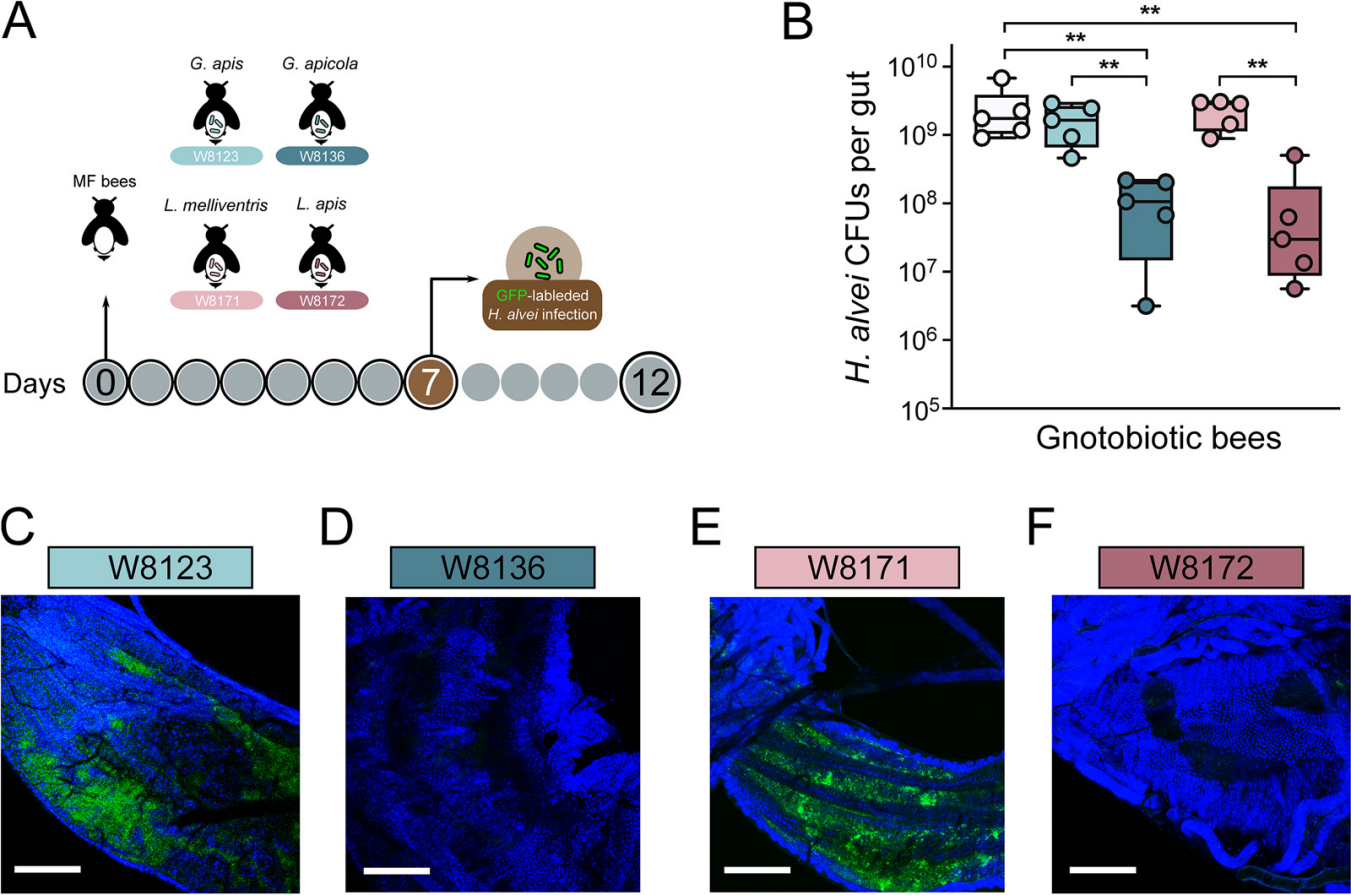
*Gilliamella* and *Lactobacillus* strains protect against *H. alvei* infection in honeybee gut. (A) Schematic illustration of experimental design. MF bees were colonized with G. apicola W8136, G. apis W8123, L. apis W8172, and L. melliventris W8171 for 7 days and then orally infected with *H. alvei* (10^6^ bacterial cells per bee). (B) Absolute abundance of H. alvei of different treatment groups 5 days postinoculation with H. alvei. Bees treated with G. apicola W8136 and L. apis W8172 showed a significant reduction of H. alvei CFU. (C–F) Confocal imaging of ileum of bees inoculated with H. alvei expressing GFP gene. H. alvei robustly colonized the ileum of bees mono-inoculated with G. apis W8123 and L. melliventris W8171 (C, E), while G. apicola W8136 and L. apis W8172 protect against H. alvei infection *in situ* (D, F). Bars = 150 μm. **, *P < *0.01 (Mann-Whitney U test).

It has been shown that honeybees with a conventional microbiome structure have higher survival following challenges with the opportunistic pathogen bacteria (11). This protection may be due to the physical block of the colonization through killing or suppressing pathogens (28) or through priming the host immune system by the gut symbionts (29). Therefore, we first determined if core bee gut members inhibit the invasion of H. alvei *in vivo*. Gnotobiotic bees mono-associated with different strains from *Gilliamella*, *Lactobacillus*, *Bartonella*, and *Bifidobacterium* were generated in the lab. MF honeybees were treated with different core gut members. Seven days postcolonization, each bee was individually fed H. alvei cell suspensions manually (10^6^ cells per bee; Fig. 2A). We found that the gut members of *Bartonella* and *Bifidobacterium* did not suppress the growth of H. alvei *in vivo* compared with the MF group (Fig. S1A in the supplemental material). However, the absolute abundance of H. alvei was significantly lower in the gut of mono-colonized bees with G. apicola W8136 and L. apis W8172 at Day 12. Interestingly, bees treated with G. apis W8123 and L. melliventris W8171 did not show a significant reduction of H. alvei (Fig. 2B). These results indicate that the ability of bee gut members in the inhibition of pathogen varied. Moreover, strains from closely related species are also different.

We then explored the pathogen resistance by gut symbionts *in situ* by visualizing the infection of engineered H. alvei expressing green fluorescence protein (GFP). We constructed H. alvei SMH01 mutant expressing GFP (H. alvei/*gfp*+; Fig. S1B–D in the supplemental material). The PA1-driven GFP gene, together with the kanamycin resistance gene, were knock-in the chromosome via homologous recombination downstream of the *lacZ* gene (Fig. S1D). H. alvei/*gfp*+ was then introduced into bees mono-associated with different bee gut strains. After 5 days of infection, we dissected the guts and explored the colonization level of the pathogen. Inspection by confocal laser scanning microscopy showed that H. alvei were tremendously cleared in guts colonized by G. apicola W8136 and L. apis W8172, while the level of H. alvei was not suppressed in the other two groups (Fig. 2C–F). These results agree with the detection by qPCR.

Alternatively, we also investigated the capacity of these gut strains to inhibit the growth of H. alvei *in vitro*. The well diffusion assay with either the liquid cultures or cell-free supernatant of bee gut strains showed that the bee gut symbionts did not inhibit the growth of H. alvei *in vitro* (Fig. S2A–D in the supplemental material). This suggests that pathogen growth was not suppressed by a direct nutrient competition or by potential antimicrobial compounds produced by the symbionts. Taken together, these findings indicate that colonization by G. apicola W8136 and L. apis W8172 suppress pathogen proliferation *in vivo*, and the resistance may be caused by the stimulation of host immune system.

### Transcriptomic responses to *Gilliamella* and *Lactobacillus* colonization.

**FIG 3:**
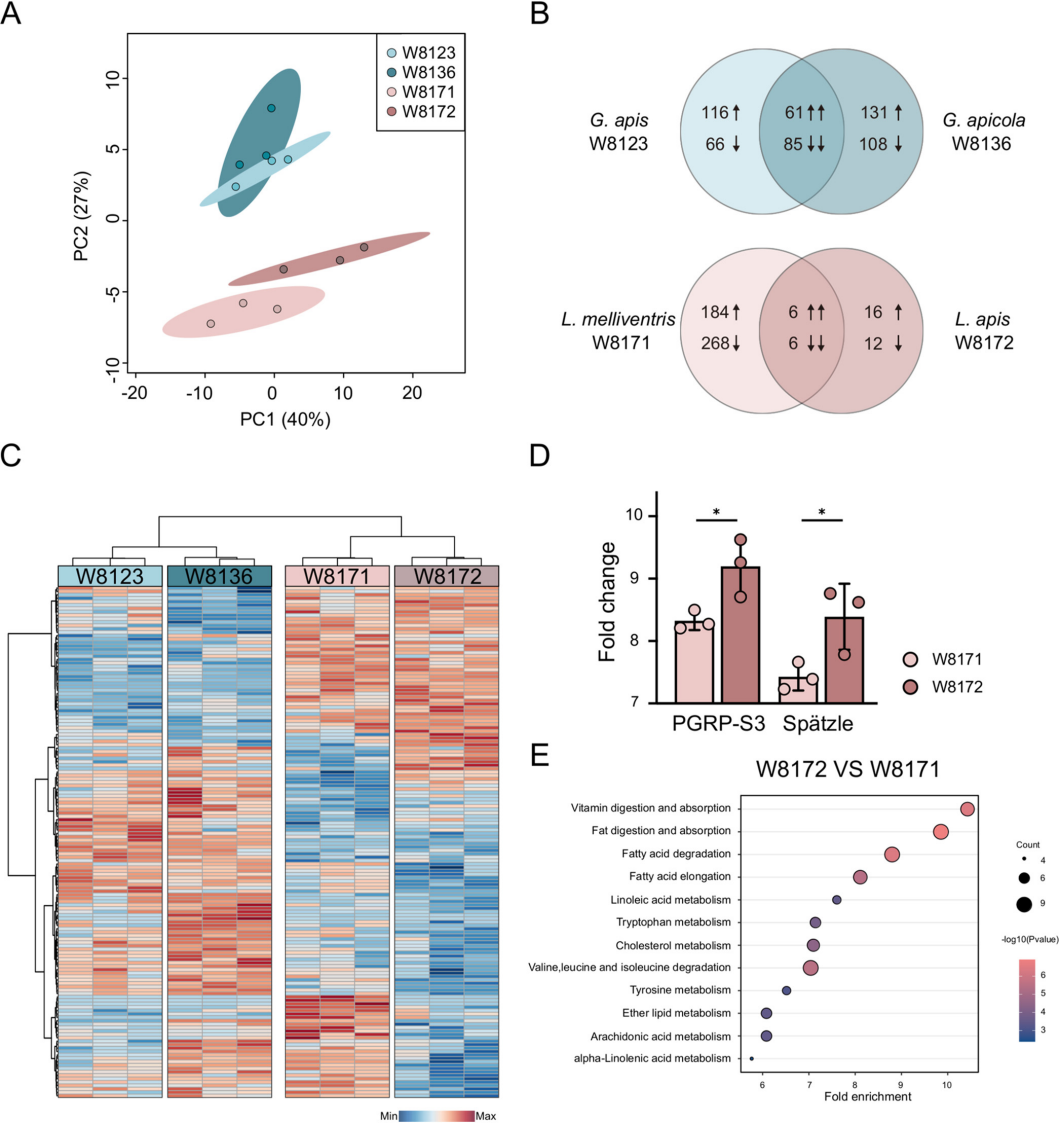
Transcriptomic analysis of the gut epithelial of honeybees mono-colonized with *Gilliamella* and *Lactobacillus* strains. (A) PCA of the RNA-seq data from the gut epithelial cells of honeybees collected from different colonization groups (*n* = 3). (B) Venn diagram representing the number of significantly differentially expressed genes (DEGs) between groups colonized with different *Gilliamella* and *Lactobacillus* strains, respectively. Numbers show the sum of up- and downregulated genes. (C) Heatmap displaying DEGs among four different colonization groups. Colors indicate the normalized relative expression of each gene (minimum-maximum). Each column represents one bee individual sample. (D) Fold change in transcript levels of the PGRP-S3 and Spätzle genes in the midgut of honeybee colonized with L. apis W8172 and L. melliventris W8171. (T-test, *P < *0.05) (E) Representative enriched KEGG pathways upregulated in the L. apis W8172 group, compared with L. melliventris W8171 group.

To better understand the host’s response to the gut symbiont colonization, we compared the transcriptional profiles of bee mono-colonized by *Gilliamella* and *Lactobacillus* strains after 24 h of inoculation. Principal coordinate analysis (PCA) showed that the two groups with different *Lactobacillus* strains were more separated, while the *Gilliamella*-colonized groups were closely related (Fig. 3A). While L. melliventris W8171 did not suppress the invasion of H. alvei, its colonization significantly changed the expression of 464 host genes (190 upregulated and 274 downregulated). L. apis W8172 colonization significantly altered the expression of 40 host genes (22 upregulated and 18 downregulated). In contrast, more genes were altered by both *Gilliamella* strains. Consistently, fewer genes were co-regulated upon colonization with *Lactobacillus* strains (six upregulated and six downregulated) than those by *Gilliamella* strains (61 upregulated and 85 downregulated) (Fig. 3B).

The top 150 significantly differentially expressed genes were plotted in a heatmap. It shows that samples inoculated with strains from the same genus clustered together, while the profiles are still separated between different strains (Fig. 3C). Although *Gilliamella* strains altered the expression of more host genes compared with L. apis W8172, immune pathways were not differentially regulated between the two tested strains. G. apicola W8136 with an inhibitory effect on *H. alvei* mainly upregulated the expression of host energy metabolism (Fig. S3 in the supplemental material). Although more host genes were altered by L. mellifera W8171, it mostly affected genes involved in the metabolism of carbohydrate, lipid, and amino acid (Fig. S4). Interestingly, among the first five significantly differentially expressed genes between the two *Lactobacillus* groups, two genes from the Toll signaling pathway were stimulated by W8172 compared with W8171. PGRP-S3 involved in the activation of Toll pathway in response to gut microbiota (30) and the cytokine-like molecule Spätzle (19) were uniquely upregulated in W8172 group.

Gene set enrichment analysis showed that several KEGG pathways were differentially regulated in the guts associated with different strains. While only few KEGG pathways were different between the two *Gilliamella*-colonizing groups (Fig. S3A in the supplemental material), more pathways were significantly upregulated by *L. apis* W8172 relative to W8171. Pathways including vitamin digestion and absorption, linoleic acid metabolism, and tryptophan metabolism were upregulated in *L. apis* W8172 gut (Fig. 3E). These findings indicated that gut bacteria impact the transcriptional profiles of the gut tissue cells. Genes involved in the metabolism and immune functions were primarily altered, which might be related to the pathogen resistance developed in the gut upon colonization with specific strains.

### *Lactobacillus* and *Gilliamella* modulate the anti-bacteria effector genes.

**FIG 4:**
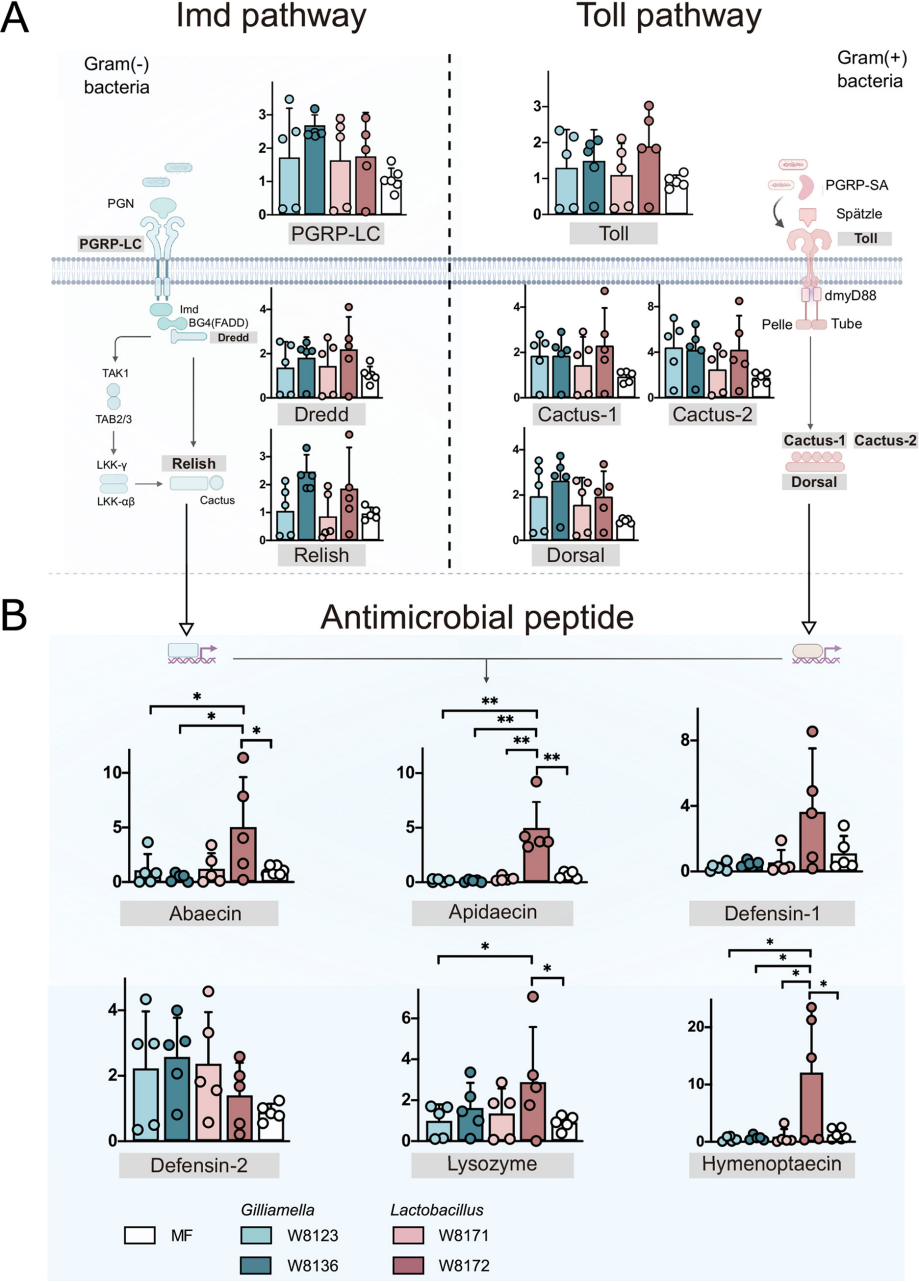
*Gilliamella and Lactobacillus* strains trigger differential host immune gene expression in Imd and Toll pathways. Gene expression was normalized relative to the housekeeping gene, *RPS18*, 24 h post-treatment. (A) Different strains did not obviously affect the expression of immune regulatory genes in the Imd and Toll pathways. (B) Mono colonization with L. apis W8172 significantly increased the expression of AMPs genes compared with the other groups. *, *P < *0.05; **, *P < *0.01 (Tukey honest method).

It has been known that gut bacteria can induce an immune response in honeybees (30). In addition, recent evidence shows that host bacterial communities are selectively regulated by the innate immune system of honeybees, and that core microbiota members demonstrate a higher level of resistance to host AMPs than opportunistic bacterial pathogens (21). Given that specific strains of G. apicola and L. apis exert anti-bacteria activity by stimulating the bee immune pathway, we further assessed the expression profiles of genes involved in immunity in mono-colonized bees (Fig. 4A). To further explore the upregulation of immune effectors following exposure to gut bacteria, we measured the relative expression of genes from Toll and Imd pathways via qPCR after inoculation with different strains. We targeted the receptors (*pgrp-lc*, *toll*), the transcription factors (*relish*, *dorsal*), and their regulators (*dredd*, *cactus*) of Imd and Toll pathways, respectively. However, no significant changes in the expression of these immune-related genes in bees inoculated with different strains and relative to the MF bees (Fig. 4A). Since we observed the difference of protection against H. alvei, we then further tested the expression of genes encoding AMPs. Interestingly, bees fed with L. apis W8172 showed increased expression of apidaecin and hymenoptaecin compared with MF bees and other mono-colonization groups (Fig. 4B). L. apis W8172-fed bees also showed higher expression of abaecin and lysozyme, but the difference was not significant compared with bees fed with L. melliventris W8171. No significant changes in other AMPs expression were detected between bees fed with G. apicola W8136 and G. apis W8123 bees for any of the AMPs tested. These results suggest that L. apis W8172 triggers an immune response with the upregulation of specific AMPs.

**FIG 5:**
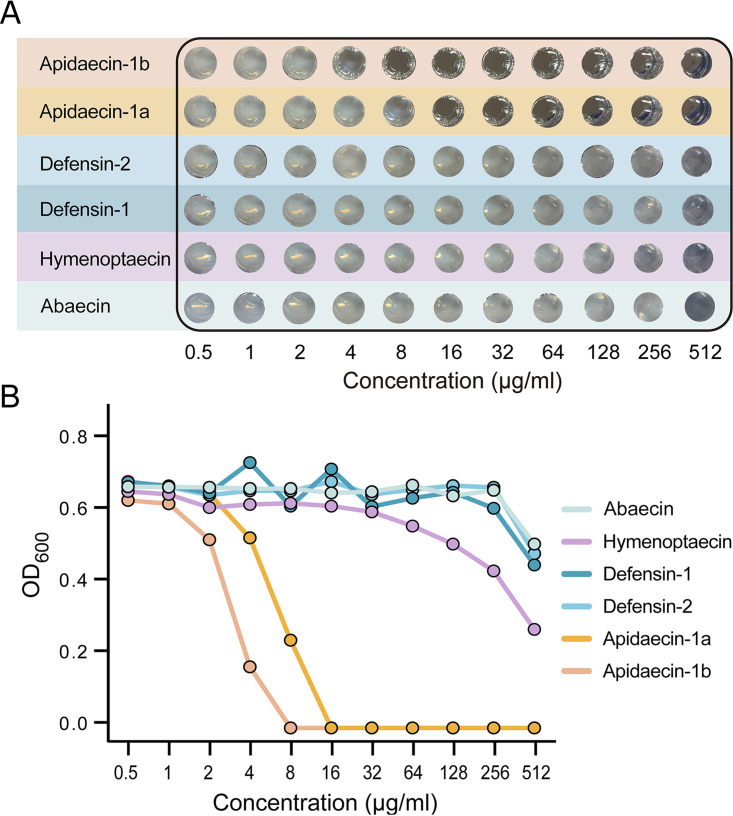
Resistance of H. alvei to different honeybee AMPs. (A) H. alvei were mainly inhibited by apidaecin-1a and 1b, while it is more resistant to abaecin, hymenoptaecin, defensin-1, and defensin-2. (B) Maximum cell density of H. alvei under different concentrations of synthesized AMPs after 24 h treatments.

To verify the inhibitory effect of AMPs secreted by honeybees on H. alvei, we performed an *in vitro* AMP resistance assay (Table S2 in the supplemental material). A. mellifera expresses two dominant apidaecin isoforms (Ia, Ib) at a ratio of approximately 1 : 20 (31). H. alvei were mainly resistant to peptides of abaecin, hymenoptaecin, defensin-1, and defensin-2 with MIC > 512 μg/ml (Fig. 5A and B). However, low concentration (8–16 μg/ml) of apidaecin-1a and 1b inhibited the growth of *H. alvei*, and the pathogen was slightly more resistant to apidaecin Ia than to Ib. This suggests that the resistance of H. alvei to AMPs is different. Specifically, two isoforms of apidaecin are more effective against the pathogen, and their *in vivo* expressions are stimulated by the Gram-positive *L. apis* strain, which also aids in the clearance of H. alvei in the gut.

### S-layer proteins unique to *Lactobacillus* as potential drivers of host immune response.

**FIG 6:**
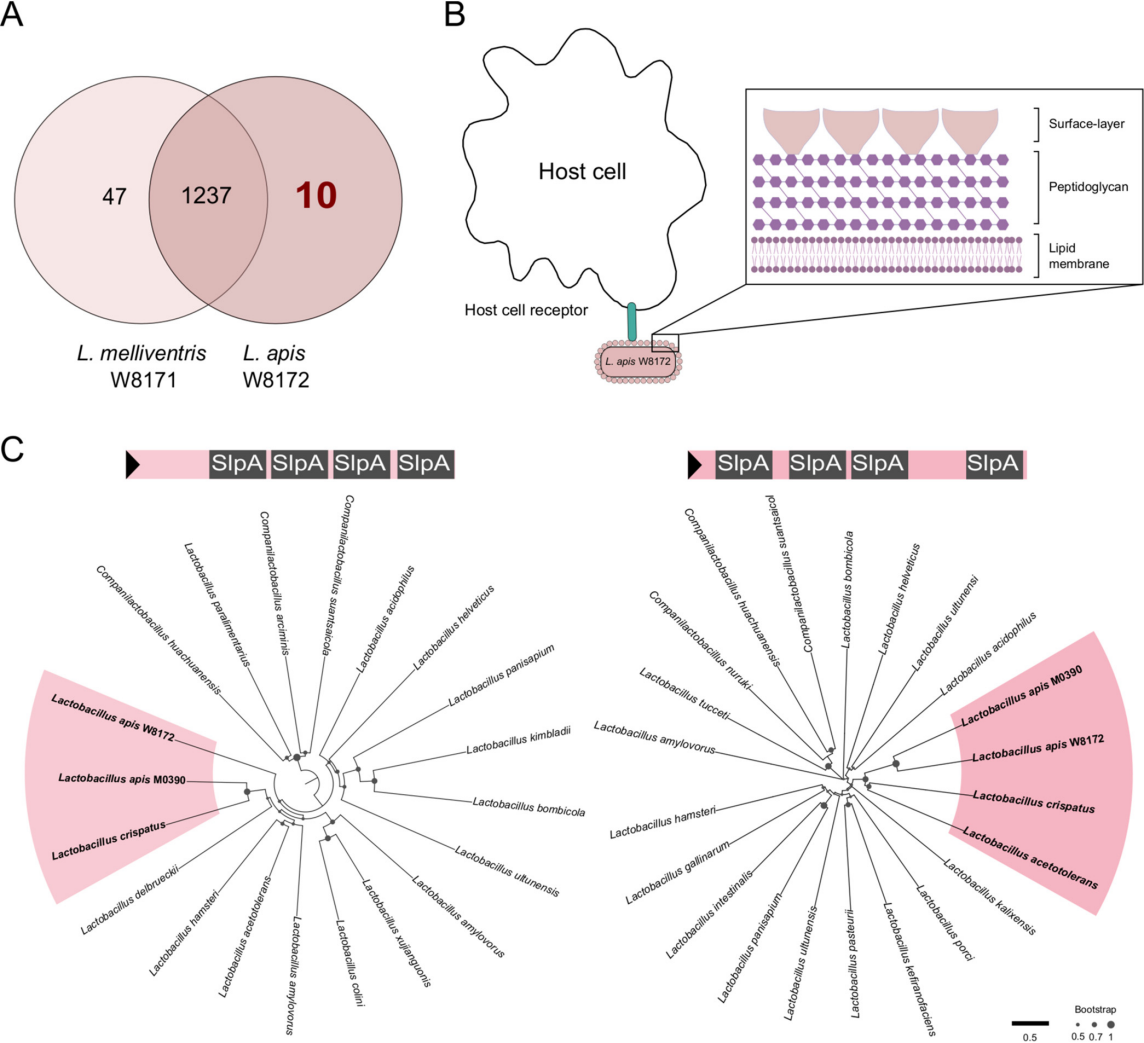
*Lactobacillus* strain W8172 with unique S-layer protein components triggers host immune system. (A) Venn diagram generated using OrthoVenn2 showing the number of shared orthologous clusters of protein-coding genes between genomes of L. melliventris W8171 and L. apis W8172. (B) Schematic illustration of the S-layer structure coating the entire cell, which facilitates the interaction with host cells and the activation of the immune response. S-layer is associated with the peptidoglycan surface of Gram-positive bacterial cell wall. (C) Neighbor-joining trees based on the amino acid sequences of S-layer proteins. Two sequences from honeybee L. apis W8172 are clustered together with that of L. crispatus from human gut.

Interestingly, the two closely related strains from *Lactobacillus* showed differences in regulating expressions of AMPs in the gut (Fig. 4B). We investigated the genes unique to L. apis W8172 but absent from L. melliventris W8171, which are potentially associated with the ability of host’s immune stimulation. The comparative analysis found that 22 genes unique to strain W8172 were enriched in 10 Gene Ontology clusters (Fig. 6A) (32). Notably, one of the enriched clusters of genes encode S-layer proteins (SLPs), which are generally formed by identical glycoprotein subunits that are held together to form a two-dimensional lattice (33). S-layer protein constitutes the outermost surface structure in those microorganisms; it is in direct contact with the bacterial environment and could mediate interactions with host cells (Fig. 6B). Due to the low amino acid sequence similarity among S-layer proteins in general, some firmicutes contain multiple S‐layer gene homologs that exhibit varying degrees of sequence identity. We identified that strain W8171 and W8172 shared three homologous SLP genes, while W8172 possessed two unique SLP genes (WP_198183937 and WP_198184146). Based on the Conserved Domain-Search analysis (34), these two genes each harbored four tandem SlpA domains (Pfam: PF03217) that have been widely detected in SLP genes of *Lactobacillus* species (Fig. 6C). Phylogenetic analysis revealed that both the orthologs of SLPs unique to W8172 were closely related to those from another strain (M0390) from L. apis isolated in honeybee gut (35). Moreover, they fell into the same radiation with the orthologs from Lactobacillus crispatus, whose SlpA is involved in the adherence to gut epithelia and potential host immune regulation (36). Thus, these two SLPs specific for L. apis strain W8172 are probably the trigger for the increased gut AMP level and the protection against pathogen H. alvei.

## DISCUSSION

This study provided experimental evidence of an interaction between honeybee gut strains and the immune system, which decreased the susceptibility to opportunistic bacterial pathogens. Honeybees with disturbed microbiota by tetracycline treatment showed decreased survivorship after the exposure to the pathogenic H. alvei, which also weakened the epithelial barrier lining the gut. Interestingly, the mono-colonization experiment showed that not all strains of the core gut members could clear H. alvei *in vivo*, and only strains from L. apis encoding specific S-layer protein stimulated the immune expression of the host. Specifically, we found a strong upregulation of the AMPs apidaecin in the guts associated with *L. apis*. The *in vitro* tests documented that H. alvei was more susceptible to the two isoforms apidaecin than the other AMPs from honeybee innate immunity. Our findings extend our previous understanding of the protection of bee gut symbionts against the bacterial pathogen and highlight a strain-level variation of gut microbes in the regulation of host immune system.

Honeybee gut bacteria form a resident community that has co-evolved with the insect host (26). In addition to playing important roles in digestion and harvesting energy (37, 38), commensal bacteria are important factors determining pathogen infection. Intestinal microbiota can confer protection against intestinal pathogens in three potential ways: (i) through indirect activation of the host immune response (39); (ii) through direct microbe-microbe competition for nutrients (39, 40); or (iii) through antagonism between microbes (e.g., via Type VI secretion system) (28, 41). In a first step toward exploring the molecular mechanism underlying the observed protection, we found that strains from *Gilliamella* and *Lactobacillus* did not inhibit the growth of H. alvei on the plates (Fig. S1 in the supplemental material), indicating that the protective effect is not provided through direct microbe-microbe interactions.

The indirect immune regulation of honeybees and other insects in response to gut commensals consists of three levels of resistance: physical barriers, cell-mediated immunity, and cell-free humoral immunity (20). Induction of honeybee innate immune responses by the gut microbes have been proposed, while only several bee gut members have been tested, such as Snodgrassella alvi (21), Lactobacillus kunkeei (23), and Frischella perrara (42). However, only S. alvi is a core member that has been repeatedly detected in individual guts of honeybees (21). Specifically, the mono colonization with S. alvi induces host AMPs and subsequently protects honeybees from the opportunistic pathogen S. marcescens. AMPs can function in the maintenance of gut microbiota homeostasis by selectively inhibiting foreign bacteria and keeping native symbionts from over proliferating (21). Here, comparing bees colonized with *L. apis* W8172 and *L. melliventris* W8171, the former had a greater upregulation of genes encoding apidaecin, abaecin, hymenoptaecin, and lysozyme (Fig. 4). Similarly, it has been shown that bees with a normal gut microbiota possess an increased level of apidaecin in the hemolymph, which facilitates the clearance of injected E. coli. It has been shown that most honeybee gut symbionts tolerate higher concentrations of apidaecin. Remarkably, the Gram-positive show MIC > 50 μg/ml of apidaecins (21). Our *in vitro* examination indicates that apidaecin is the most effective AMP to H. alvei (Fig. 5), and a concentration of apidaecin-1b down to 4 μg/ml can have a profound inhibition on the growth of H. alvei. This agrees that Gram-negative bacteria are more sensitive to apidaecins (43), and the resident gut bacteria tend to have increased tolerance of host AMPs compared with allochthonous microorganisms. Thus, the gut microbiota might activate the expression of specific AMPs that are potent to H. alvei, while the gene expression level could not fully represent the AMPs level in the body. Further Identification of the AMPs concentration induced by specific gut symbiont colonization can be identified (21). In contrast, although G. apis W8136 also assists the defense against H. alvei, it did not stimulate host immune functions but potentially energy metabolism. Indeed, *Gilliamella* is a primary polysaccharide degrader in the honeybee gut and may help detoxify specific diet sugars (27, 38), which suggests a different strategy of *Gilliamella* in pathogen clearance.

The synthesis and secretion of AMPs is a highly regulated process, which is mainly regulated by the intracellular pathways Toll and Imd (44). In *Drosophila*, bacteria activate specific recognition proteins to initiate the Toll and Imd signaling cascades (45). However, the dynamics of signaling pathway activation and the transcriptional regulation in honeybees remain unclear. Via *in silico* analyses, Lourenço et al. (46) predicted that abaecin is controlled by both the Toll and Imd pathways, whereas defensin-1 is regulated solely by Imd. Two *in vivo* experiments confirmed that abaecin and hymenoptaecin were regulated via IMD, while Dorsal is responsible for regulating defensin-1 in the Toll pathway (47). When we compared the expression of the key immune regulatory genes from both pathways, no significant changes were detected between bees with different gut strains (Fig. 4A). Likely, the mono-colonization of live cells of S. alvi did not obviously alter the expression of immune regulatory genes, while the expression of AMPs was significantly increased (29).

However, the treatment with a mixture of live and heat-killed bacterial cells triggered a more extensive immune response. Interestingly, it was shown that the gut symbionts specifically upregulated the Toll pathway compared with the Imd pathway. Our transcriptomic analysis revealed that the bees mono-colonized by L. apis W8172 showed upregulated PGRP-S3 and Spätzle in the toll pathway (Fig. 3D). Peptidoglycan recognition proteins (PGRPs) play key roles in triggering immune responses in honeybees. Honeybee harbors four short types of PGRP, including PGRP-S1, -S2, -S3, and -SA, which preferentially recognize Lys-peptidoglycan of Gram-positive bacteria (19, 48). Then, PGRP complexes cleave the Spätzle precursor into a mature form, which binds to the Toll protein and initiates the transcription of AMPs (49, 50). Therefore, our results suggest that the cell wall component of the L. apis strain triggers the pattern recognition receptor of the host and leads to the downstream immune response (51).

However, strain W8171 from L. melliventris did not induce the PGRP and Spätzle signal transduction. Genomic comparison finds that the two species are mainly different in the S-layer protein, which is a part of the cell envelope identified in almost all bacteria (Fig. 6A). It has been documented that S-layer proteins perform as adhesion medium binding to the intestinal epithelial cells and mucus, such as surface-protein from Lactobacillus plantarum strains 423, AR326, and AR269 (52, 53). We found that two genes encoding the S-layer protein are only present in the genome of W8172. The phylogenetic analysis revealed that the two genes were closely related to L. crispatus, respectively (Fig. 6C). S-layer proteins are responsible for gut bacteria to adhere to host cells and competitively to exclude pathogens from invasion (54). Moreover, the S-layer proteins can activate host’s innate immune system, which is mediated by the recognition through the Toll pathway (55). Consistently, we found that W8172 induced the expression of PGRP-S3 and Spätzle, two key regulators of the Toll pathway in honeybee immune system (Fig. 3D). Thus, our results suggest the important roles of S-layer proteins in L. apis strains in recognizing the host’s immune system. A plasmid toolkit has been developed for engineering bee gut Proteobacteria (56), whereas a suitable Gram-positive plasmid is still lacking for the genetic manipulation of bee gut-specific *Lactobacillus* strains. Targeted disruption of genes encoding S-layer proteins in L. apis strains will allow the investigation of the effect on the immune stimulation and the suppression of H. alvei in the gut.

Pathogens are considered one of the largest contributing factors in the global decline of honeybees. H. alvei strains are widespread in honeybee guts, and our results confirmed their lethal infections. Although the native gut symbionts are important in the resistance to the pathogen invasion, our results indicated the variation of different strains in the regulation of host immune functions. Multiple species have been identified within all the core gut members of honeybees, and further strain-level variations have also been reported within each species (57). Our findings highlight the important role of gut microbiota in controlling bee pathogens and the mechanism underlying the difference of strains in host’s immune regulation. Thus, microbial-based therapeutics may offer a simple but effective solution to reduce honeybee disease burden, environmental xenobiotic exposure, and the spread of antimicrobial resistance.

## MATERIALS AND METHODS

### Isolation and genome sequencing of bacteria.

H. alvei strain SMH01 and honeybee gut bacterial strains were isolated from the guts of A. mellifera collected in Jilin, China, during July 2018. The dissected guts were directly crushed in 20% (vol/vol) glycerol and frozen at −80°C after sampling. The glycerol stocks were plated on heart infusion agar (HIA) supplemented with 5% (vol/vol) defibrinated sheep’s blood (Solarbio, Beijing, China), MRS agar (Solarbio), or TPY agar (Solarbio) incubated at 35°C under a CO_2_-enriched atmosphere (5%). Purified isolates were then amplified using PCR with universal bacterial primers 27F and 1492R. The detailed information of all isolates is listed in Table S3 in the supplemental material. The 16S rRNA sequence of *H. alvei* strain SMH01 was deposited in NCBI (GenBank Acc. No. OK2068159).

### H. alvei infection survival assay.

Approximately 200 adult worker bees were collected from brood frame from the hive kept at Miyun County, Beijing (March 2019). The bees were immobilized at 4°C and were divided into two cohorts (control and tetracycline treatment) distributed to cup cages as previously described (11). The cup cages were maintained in an incubator at 35°C, with humidity of 50%. Each cohort has five replicating cup cages containing ∼20 bees in each cup. Control groups were fed filter-sterilized 0.5 M sucrose syrup, and treatment bees were fed 450 μg/ml of tetracycline suspended in the sucrose syrup. After 3 days, the tetracycline treatment was stopped, and the bees were randomly redistributed into four experimental groups with ∼30 bees from each group: (i) control; (ii) control+*H. alvei*; (iii) tetracycline treatment (Tet); and (iv) Tet+*H. alvei*. Control and Tet groups were fed only sterile sugar syrup and pollen grains, while the other two groups were infected with *H. alvei.* For the groups infected by *H. alvei*, the bees were feeding on the corresponding suspensions for 24 h. Bacterial cells of *H. alvei* SMH01 resuspended in 1 ml 1×PBS at a final concentration of 3 × 10^8^ CFU/ml were mixed with 1 ml sterilized sucrose solution and 0.3g irradiated pollen grains.

### Antibacterial assay of the honeybee gut symbionts against H. alvei.

G. apis W8123, G. apicola W8136, B. apousia W8102, and B. apis W8152 isolated from honeybee guts, and H. alvei SMH01 (Table S3 in the supplemental material) were grown on HIA supplemented with 5% (vol/vol) sterile sheep blood (Solarbio). L. melliventris W8171 and L. apis W8172 were grown on MRS agar plates (Solarbio) supplemented with 0.1% L-cystine and 2.0% fructose. Cell suspensions of bee gut bacteria was prepared by collecting colonies and diluting to a final concentration of ∼10^8^ CFU/ml in 1×PBS. The cell-free supernatant was obtained by centrifuging bacterial suspensions at 5000 × *g* for 5 min, followed by filtration through a 0.22-µm pore size syringe filter (Minisart 16532-K, Sartorius, Göttingen, Germany). H. alvei was grown on HIA and incubated overnight. Colonies were collected from the plates and were diluted to 10^6^ CFU/ml using 1×PBS. Then, 100 µl of the H. alvei culture suspension was streaked over the surface of the Columbia agar plates and spread using a glass spreader. A well with 10-mm diameter size was made in the middle of the agar plate, and 100 μl of the supernatants were put into the well. The plates were incubated at 35°C and 5% CO_2_ for 48 h.

### Bees mono-colonized with gut symbionts challenged with H. alvei.

To visualize the inhibition of H. alvei by bee gut strains *in vivo*, we engineered H. alvei SMH01 mutant constitutively expressing the green fluorescent protein. We designed homologous recombination plasmid parts based on the Bee Microbiome Toolkit (56). Three new parts were prepared: (i) a Type 2 part with upstream region of the chromosomal *lacZ* gene, (ii) a Type 3 part containing a *KanR* sequence and *GFP* sequence, and (iii) a Type 4 part with downstream region of the chromosomal *lacZ* gene. Golden Gate assembly of these new parts with the other parts pYTK002 (Type 1), pYTK072 (Type 5), pBTK301 (Type 6–7), and pBTK401 (Type 8) was performed as previously described (56) to assemble complete plasmids with GFP. Then the constructed plasmid was connected with PBTK599s using BsmBI (New England Biolabs, MA, USA) to construct a suicide plasmid vector. The plasmid was electroporated into E. coli donor strain MFD*pir* for conjugation.

MF bees were obtained as described by Zheng et al. (38). Late-stage pupae were removed manually from brood frames and placed in sterile plastic bins. The pupae emerged in an incubator at 35°C, with humidity of 50%. Newly emerged MF bees (Day 0) were kept in axenic cup cages with sterilized sucrose syrup for 24 h. For each mono-colonization setup, 20∼25 MF bees were placed into one cup cage, and the bees were feeding on the bacterial culture suspensions for 24 h. For the MF group, 1 ml of 1×PBS was mixed with 1 ml of sucrose solution and 0.3 g sterilized pollen. For the other group, glycerol stock of bee gut strains was resuspended in 1 ml 1×PBS at a final concentration of ∼10^8^ CFU/ml, and then mixed with 1 ml sterilized sucrose solution. All bees were kept in an incubator (35°C, RH 50%) until Day 7.

To precisely control the infection amount of *H. alvei* cells, bees from the mono-colonization and MF groups were inoculated with *H. alvei* SMH01/*gfp*+ individually by oral feeding. Cell suspensions of *H. alvei*/*gfp*+ were prepared by collecting colonies from plates into 20% sucrose in 1×PBS. Each bee individual that had been starved for 3 h was fed with 5 μl of the cell suspension. Inoculum levels of *H. alvei* were ascertained by enumerating CFU from plated serial dilutions of the cell suspension. Each bee individual was fed exactly 1 × 10^6^ CFU of *H. alvei*/*gfp*+. After 5 days, the load of *H. alvei* was determined by qPCR.

### qPCR determining H. alvei loads in gut samples.

DNA was extracted from gut homogenates using CTAB method (58). DNA concentration was determined with Qubit 4 Fluorometer (Thermo Fischer Scientific; Waltham, MA, USA). H. alvei loads were determined by qPCR using the ChamQ Universal SYBR qPCR Master Mix (Vazyme Biotech, Nanjing, China). H. alvei-specific primer sets are listed in Table S1 in the supplemental material. All qPCRs were performed in 96-well microplates on a QuantStudio 1 real-time PCR system (Thermo Fischer Scientific). Melting curves were generated after each run (95°C for 15 s, 60°C for 20 s, and increments of 0.3°C until reaching 95°C for 15 s). Standards for *H. alvei* 16S rRNA and the host’s actin were prepared by cloning sequences into the pCE2 TA/Blunt-Zero Vector (Vazyme Biotech). Each reaction was performed in triplicates on the same plate. The data was analyzed using the QuantStudio Design and Analysis Software. After calculating H. alvei 16S rRNA gene copies, normalization was performed to reduce the effect of gut size variation and extraction efficiency using the host’s actin gene (59).

### Visualizing fluorescent bacteria *in situ*.

Fluorescent images were obtained on a confocal laser scanning microscopy (A1CLSM; Nikon, Tokyo, Japan). Bees were chilled, and the guts were dissected. Without punctuation, entire gut compartments were transferred to the Ibidi μ-Dish 35 mm and then placed on the microscope. Images were taken with a 20× objective.

### Transcriptomic analysis.

Three-day-old MF bees were mono-colonized with G. apicola W8136, G. apis W8123, L. apis W8172, and L. melliventris W8171. Then, 24 h after the colonization, whole guts were dissected in ice-cold PBS, and total RNA was extracted using the Quick-RNA MiniPrep kit (Zymo Research). RNA was eluted in 50 μl of RNase-free water and stored at −80°C. RNA sequencing libraries were generated using NEBNext Ultra RNA Library Prep Kit for Illumina (New England BioLabs). The clustering of the index-coded samples was performed on a cBot Cluster Generation System using TruSeq PE Cluster Kit v3-cBot-HS (Illumina; San Diego, CA, USA), and the library preparations were then sequenced on an Illumina NovaSeq 6000 platform, and 150 bp paired-end reads were generated. Gene expression was quantified using HTSeq v0.7.2 (https://doi.org/10:1093/bioinformatics/btu638). Differential expression analysis was performed using the DESeq2 package (60). Functional analysis of DEGs was performed based on KEGG Orthologue markers using clusterProfiler (https://doi.org/10.1089/omi.2011.0118). Raw RNA-seq data have been deposited at NCBI SRA database under the BioProject accession number PRJNA767946.

To further determine host gene expression, quantitative reverse transcription-PCR analysis was performed with the PikoReal 96 (Thermo Fischer) using the qPCR SYBR green Master Mix (Vazyme). Total RNA of each dissected gut was extracted as described above. cDNA was then synthesized using the HiScript III RT SuperMix for qPCR (Vazyme). qPCR was performed using the ChamQ Universal SYBR qPCR Master Mix (Vazyme) and QuantStudio 1 Real-Time PCR Instrument (Thermo Fisher Scientific) in a standard 96-well block. The housekeeping *RPS18* gene was chosen as the inner reference gene, and relative expression was determined using the 2-ΔΔCT method (21). The primers for different host genes are shown in Table S1 in the supplemental material.

### Antimicrobial peptide resistance assays.

To test the activity of AMPs against *H. alvei*, growth inhibition assays in liquid media were performed using 50% diluted HIA media. AMPs synthesized by GL Biochem and Sangon Biotech were serially diluted in 96-well plates to obtain concentrations of 0.5 to 512 µg/ml. Overnight grown H. alvei cultures from HIA media were diluted to 1 × 10^6^ CFU/ml in 50% Heart Infusion Broth. Optical density was recorded after 24 h of incubation at 35°C, 5% CO_2_.

### Data availability.

Raw RNA-seq data have been deposited at NCBI SRA database under the BioProject accession number PRJNA767946.
