# Supplementary material for: Specific Strains of Honeybee Gut Lactobacillus Stimulate Host Immune System to Protect against Pathogenic Hafnia alvei
Source: Microbiol Spectr. 2022 Jan 5;10(1):e01896-21. doi: 10.1128/spectrum.01896-21 (PMC8729767; doi:10.1128/spectrum.01896-21)
Supplement: SUPPLEMENTAL FILE 1 — Supplemental material. Download SPECTRUM01896-21_Supp_1_seq8.pdf, PDF file, 1.7 MB [file spectrum01896-21_supp_1_seq8.pdf]

Supplemental Material

For: Specific strains of honeybee gut *Lactobacillus* stimulate host immune system to protect against pathogenic *Hafnia alvei*

Haoyu Lang, Huijuan Duan, Jieni Wang, Wenhao Zhang, Jun Guo, Xue Zhang, Xiaosong Hu, Hao Zheng,

**Contents:**	**Page**
Figure S1	2
Figure S2	3
Figure S3	4
Figure S4	5
Table S1	6
Table S2	7
Table S3	8
Supplementary References	9

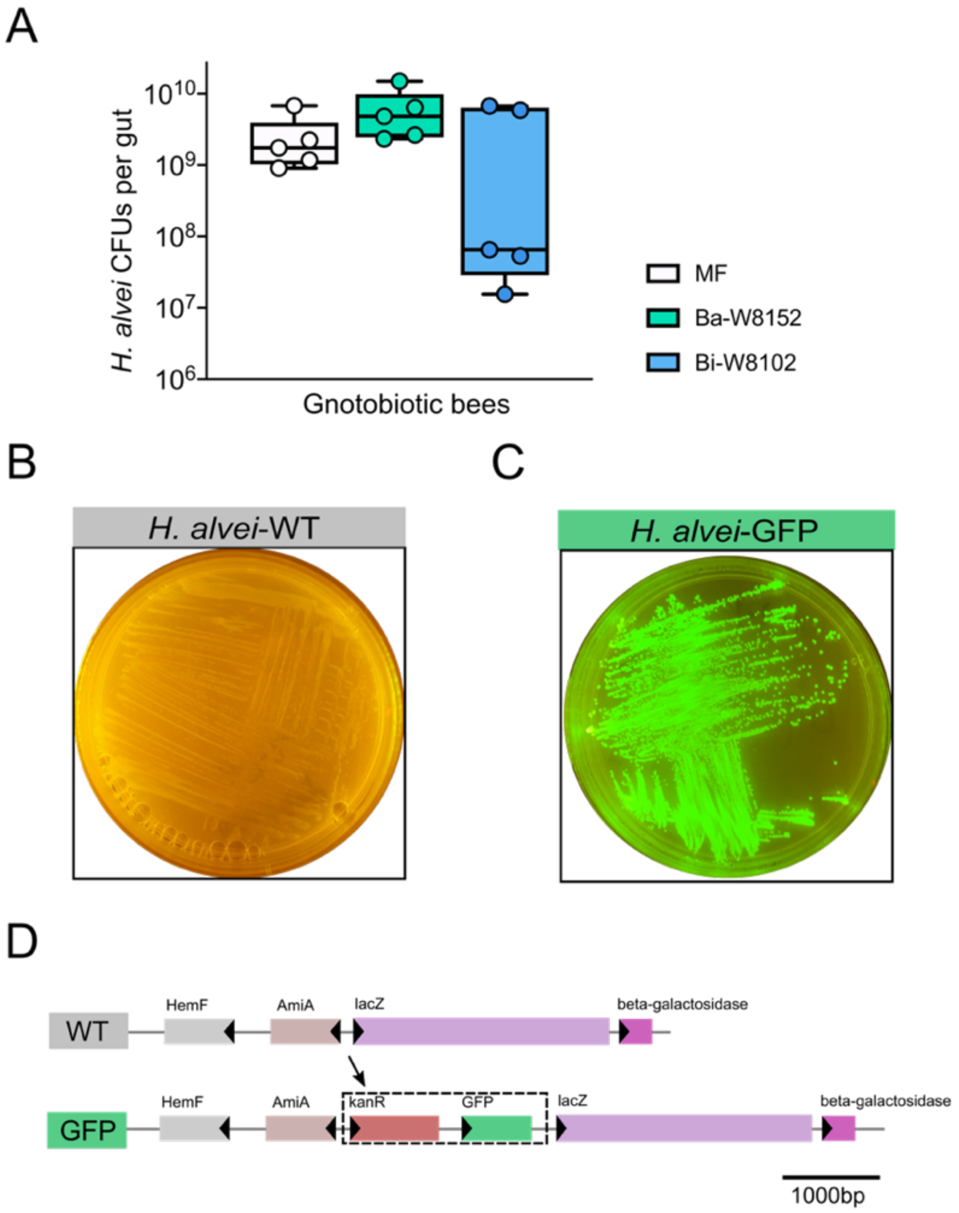

FIG S1 *B. apousia* and *B. apis* strains did not protect against *H. alvei* infection in honeybee gut, and scheme for engineered *H. alvei* SMH01 mutant constitutively expressing the green fluorescent protein. (A) Absolute abundance of *H. alvei* of different treatment groups 5 days post-inoculation with *H. alvei*. Bees treated with *B. apousia* W8102 and *B. apis* W8152 do not affect the colonization of *H. alvei*. (B) Wild-type *H. alvei.* (C) Green fluorescent mutant *H. alvei.* (D) The GFP gene and the kanamycin resistance gene were knocked-in the chromosome downstream of the *lacZ* gene.

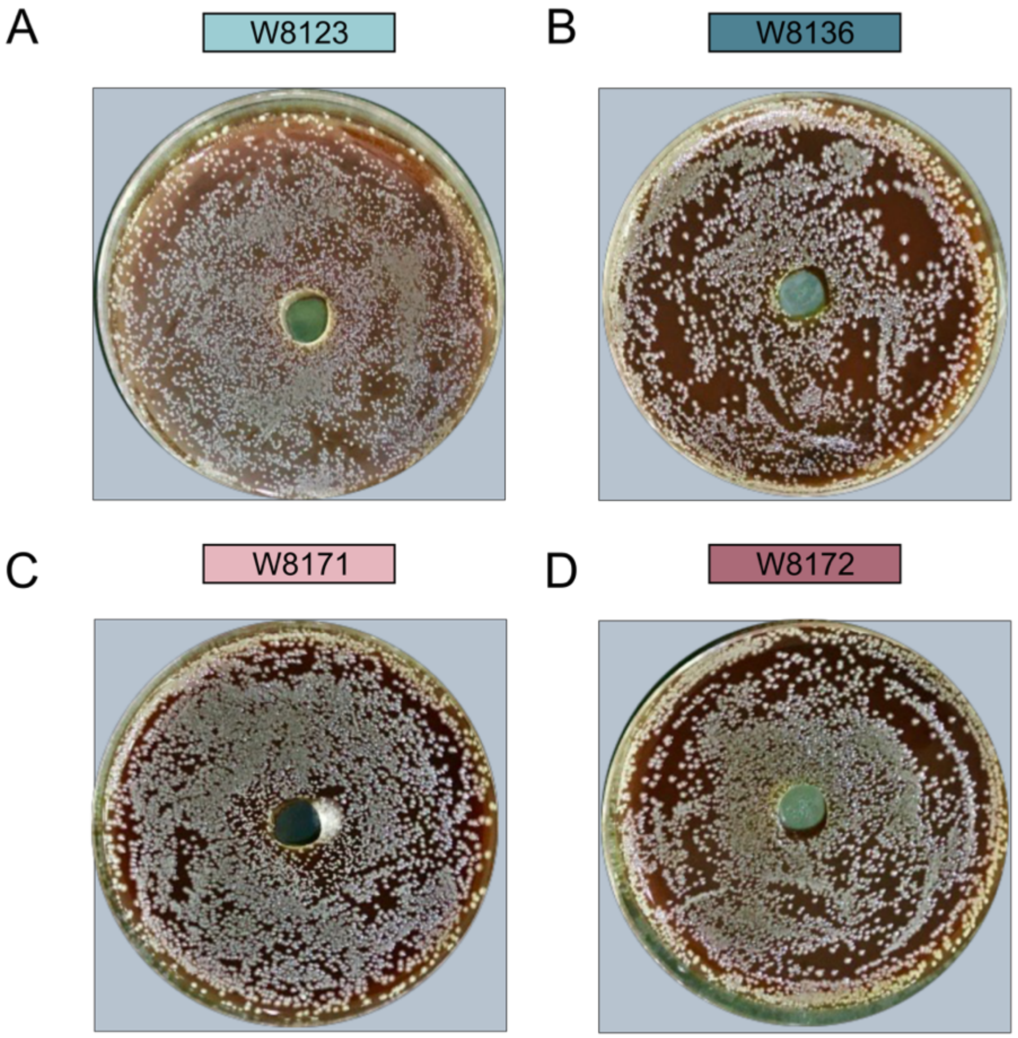

FIG S2 *Gilliamella* and *Lactobacillus* strains did not protect against *H. alvei* infection in the honeybee gut. (A-D) The liquid cultures or cell-free supernatant of *G. apicola* W8136, *G. apis* W8123, *L. melliventris*, and *L. apis* W8172 did not inhibit the growth of *H. alvei in vitro*.

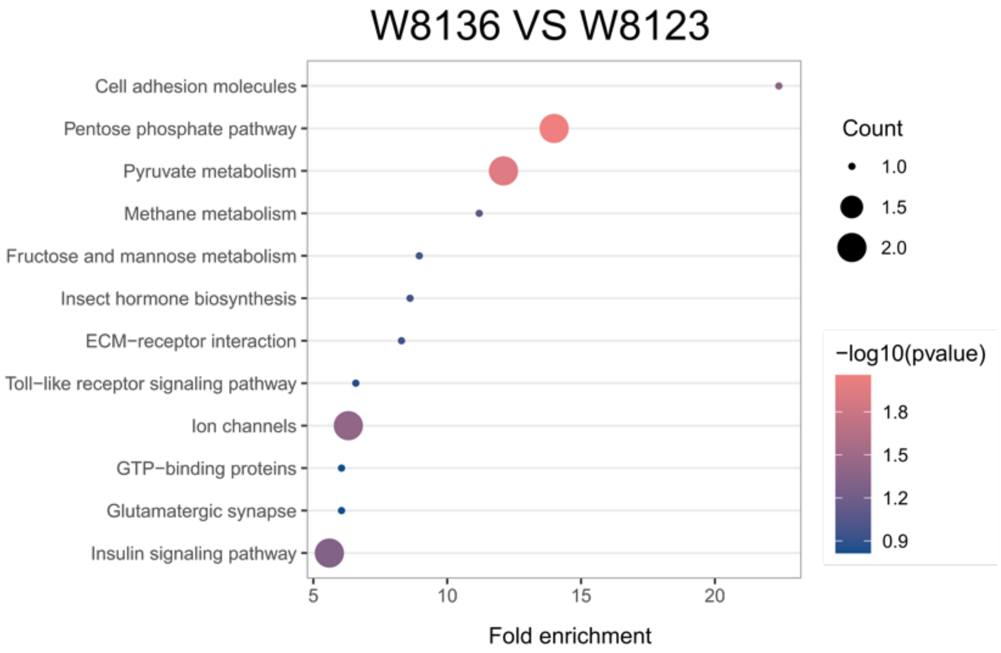

FIG S3 KEGG analysis of the gut epithelial of honeybees mono-colonized with *Gilliamella*. Representative enriched KEGG pathways upregulated in the *G. apicola* W8136 group, compared to W8123.

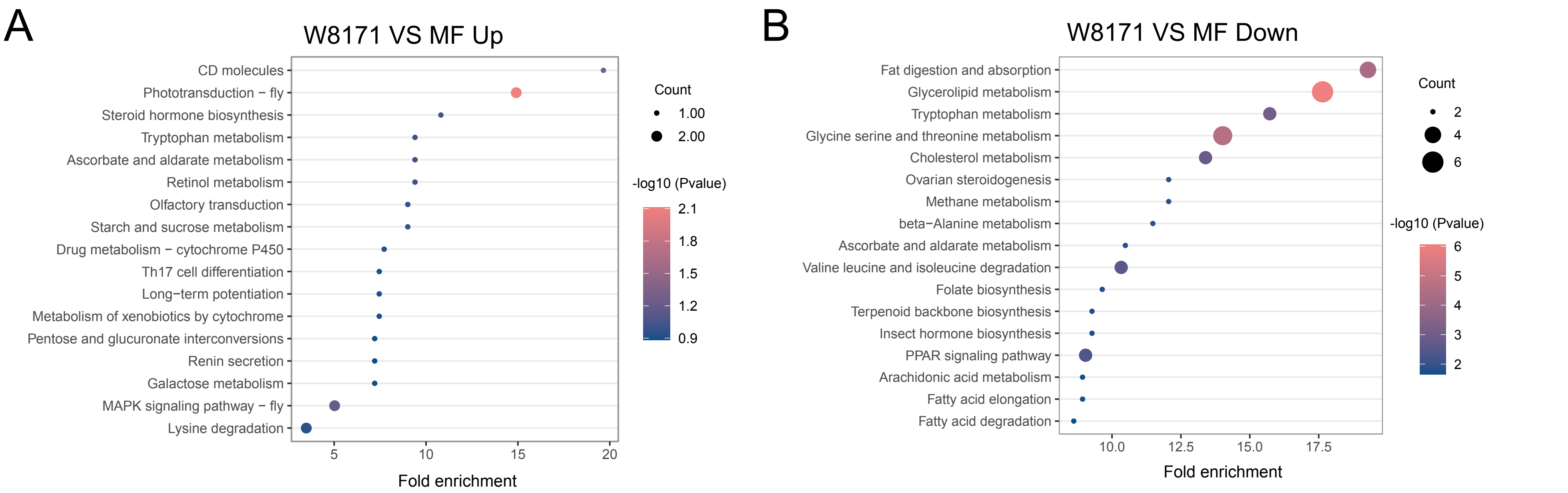

FIG S4 KEGG analysis of the gut epithelial of honeybees mono-colonized with *L. melliventris* W8171. Representative enriched KEGG pathways up- (A) and down-regulated (B) in the *L. melliventris* W8171 group, compared to MF.

Table S1. qPCR primer sequences for gene expression analysis.

Gene	Reference	Forward	Reverse
Abaecin	(1)	TCGGATTGAATGGTCCCTGAC	ATCTTCGCACTACTCGCCAC
Apidaecin	(1)	GTAGGTCGAGTAGGCGGATCT	TTTTGCCTTAGCAATTCTTGTTG
Cactus-1	(1)	CTATCGTGGAGAAACTGCGTAT	TCAGGAAGTGGTTCTGGTATTG
Cactus-2	(1)	ATCAGACGGCTCTGCTCTAT	TCGTCTTCGTCAGTGGTATCT
Dorsal	(1)	AGAGATGGAACGCAGGAAAC	TGACAGGATATAGGACGAGGTAA
Dredd	(1)	GCGTCATAAAGAAAAAGGATCA	TTTCGGGTAATTGAGCAACG
Hymenoptaecin	(1)	GTCGTCCATCCTTGGACATT	TTTCCCAAACTCGAATCCTG
PGRP-LC	(1)	TCCGTCAGCCGTAGTTTTTC	CGTTTGTGCAAATCGAACAT
Relish	(1)	GGAGCTGATCCAAATCGAAC	AGTGGCATCCATCCATCATT
RPS18	(1)	AGGTGTTGGTCGTCGTTAT	CATTCTCCAGCACGCTTAT
Toll	(1)	TAGAGTGGCGCATTGTCAAG	ATCGCAATTTGTCCCAAAAC
Defensin-1	(2)	TGCGCTGCTAACTGTCTCAG	AATGGCACTTAACCGAAACG
Defensin-2	(3)	GCAACTACCGCCTTTACGTC	GGGTAACGTGCGACGTTTTA
Lysozyme	(3)	ACACGGTTGGTCACTGGTCC	GTCCCACGCTTTGAATCCCT
Actin	(4)	TGCCAACACTGTCCTTTCTG	AGAATTGACCCACCAATCCA

Table S2. AMPs used in this study.

AMPs	Sequence	Length	Reference
abaecin	YVPLPNIPQPGRRPFPTFPGQGPFNPKIKWPQGY	34	(5)
apidaecin-1a	GNNRPVYIPQPRPPHPRI	18	(6)
apidaecin-1b	GNNRPVYIPQPRPPHPRL	18	(6)
defensin-1	VTCDLLSFKGQVNDSACAANCLSLGKAGGHCEKGVCICRKTSFKDLWDKRFG	52	(7)
defensin-2	VTCDVLSWQSKWLSINHSACAIRCLAQRRKGGSCRNGVCICRK	43	(7)
hymenoptaecin	QERGSIVIQGTKEGRNRPSLDIDYKQRVYDKNGMTGDAYGGVNIRPGQPTRQHA<br>GFEFGKEYKNGFIRGQSEVQRGPGGRLSPYVGINGGFRF	93	(8)

Table S3. Bacterial isolates used in this study.

Strain	Collection_site	Isolated medium	NCBI accession number
Hafnia alvei SMH01	China: Jilin	Heart infusion agar	OK206815 (16S rRNA sequence)
Bartonella apis W8152	China: Jilin	Heart infusion agar	GCF_016100395
Bifidobacterium asteroids W8102	China: Jilin	Heart infusion agar	GCF_007559275
Gilliamella apis W8123	China: Jilin	Heart infusion agar	GCF_016101085
Gilliamella apicola W8136	China: Jilin	Heart infusion agar	GCF_016101285
Lactobacillus melliventris W8171	China: Jilin	MRS	GCF_016102065
Lactobacillus apis W8172	China: Jilin	MRS	GCF_016102055

Supplementary References

1. Horak R D, Leonard S P, Moran N A. Symbionts shape host innate immunity in honeybees. Proc Biol Sci. 2020; 287(1933):20201184.
2. Daisley B A, Pitek A P, Chmiel J A, Al K F, Chernyshova A M, Faragalla K M, et al. Novel probiotic approach to counter Paenibacillus larvae infection in honey bees. ISME J. 2020; 14(2):476-491.
3. Daisley B A, Pitek A P, Chmiel J A, Gibbons S, Chernyshova A M, Al K F, et al. Lactobacillus spp. attenuate antibiotic-induced immune and microbiota dysregulation in honey bees. Commun Biol. 2020; 3(1):534.
4. Kesnerova L, Emery O, Troilo M, Liberti J, Erkosar B, Engel P. Gut microbiota structure differs between honeybees in winter and summer. ISME J. 2020; 14(3):801-814.
5. Casteels P, Ampe C, Riviere L, Van Damme J, Elicone C, Fleming M, et al. Isolation and characterization of abaecin, a major antibacterial response peptide in the honeybee (Apis mellifera). Eur J Biochem. 1990; 187(2):381-386.
6. Welch N G, Li W, Hossain M A, Separovic F, O'Brien-Simpson N M, Wade J D. (Re)Defining the proline-rich antimicrobial peptide family and the Identification of putative new members. Front Chem. 2020; 8:607769.
7. Klaudiny J, Albert S, Bachanova K, Kopernicky J, Simuth J. Two structurally different defensin genes, one of them encoding a novel defensin isoform, are expressed in honeybee Apis mellifera. Insect Biochem Mol Biol. 2005; 35(1):11-22.
8. Casteels P, Ampe C, Jacobs F, Tempst P. Functional and chemical characterization of Hymenoptaecin, an antibacterial polypeptide that is infection-inducible in the honeybee (Apis mellifera). J Biol Chem. 1993; 268(10):7044-7054.
